# Can pressurised intraperitoneal aerosol chemotherapy with oxaliplatin (PIPAC-O+) be added to standard treatment for resectable high-risk gastric cancer patients? A study protocol

**DOI:** 10.1515/pp-2021-0132

**Published:** 2021-09-17

**Authors:** Jessica L. Reid, Harsh A. Kanhere, Peter J. Hewett, Timothy J. Price, Guy J. Maddern, Markus I. Trochsler

**Affiliations:** Discipline of Surgery, The University of Adelaide, The Queen Elizabeth Hospital, Adelaide, SA, Australia; Department of Surgery, Royal Adelaide Hospital, Adelaide, SA, Australia; Department of Oncology, The Queen Elizabeth Hospital, Adelaide, SA, Australia; Adelaide Medical School, The University of Adelaide, Adelaide, SA, Australia

**Keywords:** gastric, PIPAC, proof of concept

## Abstract

**Objectives:**

Gastric cancer remains one of the most fatal cancers, despite an intensive treatment regime of chemotherapy–surgery–chemotherapy. Peritoneal metastatic disease is commonly diagnosed post treatment regime and once established, patients are likely to die in 3–9 months. Systemic chemotherapy does not increase survival for these patients due to the poor vascularisation of this area. We are proposing the addition of pressurised intraperitoneal aerosol chemotherapy (PIPAC) to the treatment regime for curative patients as a preventive measure to reduce the risk of peritoneal metastases occurring.

**Methods:**

This is a prospective, single centre, non-randomised, open-label pilot trial evaluating the addition of PIPAC to the standard multimodal treatment pathway. Patients will undergo standard neoadjuvant chemotherapy with four cycles of fluorouracil, leucovorin, oxaliplatin and docetaxel (FLOT), then PIPAC, followed by gastrectomy. Four cycles of FLOT will be administered post-surgery. Primary outcome is safety and feasibility, assessed by perioperative morbidity and possible interruptions of the standard multimodal treatment pathway.

## Introduction

Gastric cancer is the fourth most frequent cancer worldwide and the third and fifth most frequent cancer related cause of death in men and women, respectively [1]. While the incidence of gastric adenocarcinoma has declined, there is a trend to more proximal and biologically more aggressive variants such as diffuse signet cell types [2]. Prognosis remains dismal with an overall 5-year survival of 25% for all stages. Surgical resection remains the mainstay of curative treatment. The addition of modern pre- and post-operative chemotherapy has improved progression free and overall survival for stage II and III patients of which FLOT (fluorouracil, leucovorin, oxaliplatin and docetaxel) is the standard protocol frequently used in non-Asian populations [3]. Despite this multimodal approach these patient groups still show a low 5-year survival of up to 40% with a recurrence rate of 40–80% [4].

Peritoneum is the most frequent site of metastatic disease in gastric cancer patients [5]. Once peritoneal metastatic disease is established, life-expectancy is limited to about 3–9 months [5]. Systemic chemotherapy has not been able to provide a significant survival benefit for these patients as drug distribution to the peritoneum is restricted by vascular supply [6]. Therefore, preventing peritoneal disease potentially improves progression free and overall survival of gastric cancer patients treated with curative intent.

It is well accepted that gastric cancer patients with higher *T* stage, nodal involvement, linitis plastica, younger age, proximal gastric location and positive cytology have an increased risk for developing peritoneal recurrent disease [7]. There is evidence to suggest that intraperitoneal chemotherapy in high risk gastric cancer improves one-, two- and three-year overall survival with a reduction in the rates of peritoneal recurrence and distant metastasis although these results do vary and further advances are required [8, 9].

### PIPAC

PIPAC was developed as a method of delivering chemotherapy directly and more efficiently to the peritoneal cavity. Better pharmacokinetic distribution and penetration of the drug within the peritoneal cavity by this technique combined with minimal systemic side effects have led to a global uptake of PIPAC in the palliative treatment of peritoneal gastric cancer metastasis [4, 5, 10]. More importantly, multiple studies collated by Alberto et al. have shown a beneficial safety profile and low numbers of adverse events [1]. No data is currently available on the use of PIPAC in as peri-operative treatment of high-risk gastric cancer without macroscopic peritoneal metastasis. A prospective safety trial in two Danish and Swedish hospitals is in preparation to evaluate the use of PIPAC at the time of gastric surgery in high risk gastric cancer patients [11].

### Aim

The aim of this study is to assess safety and feasibility of adding PIPAC to the standard FLOT pathway for high risk gastric adenocarcinoma patients.

The investigators have termed this as PIPAC-O+ (PIPAC with Oxaliplatin) reflecting the addition of PIPAC with oxaliplatin to the standard multimodal treatment concept for non-stage IV gastric cancer patients with high-risk features.

The intention is to provide a suitable pathway for delivering chemotherapy directly to the peritoneum to reduce the risk of peritoneal metastases, in addition to the current gold standard therapy of peri-operative systemic FLOT chemotherapy and gastrectomy.

The primary objective of safety and feasibility will be assessed by perioperative morbidity and possible interruptions of the standard multimodal treatment pathway for these patients. Secondary objectives to evaluate are conversion of lavage cytology, perioperative status and morbidity associated to the trial intervention, progression free survival, overall survival and quality of life.

## Materials and methods

### Study design

This is a prospective, single centre, non-randomised, open-label pilot trial evaluating the addition of PIPAC-O+ to the standard multimodal treatment pathway. The overall duration of the trial is expected to be approximately three years.

**Figure 1:**
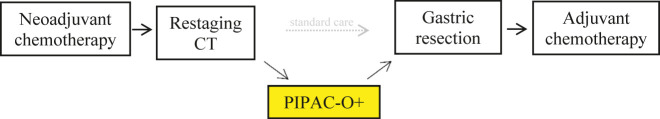
PIPAC-O+ pathway. Patients will follow the standard FLOT protocol, with Oxaliplatin administered via PIPAC prior to gastric resection.

Patients will undergo standard neoadjuvant chemotherapy with four cycles of fluorouracil, leucovorin, oxaliplatin and docetaxel (FLOT), then PIPAC-O+, followed by gastrectomy. Four cycles adjuvant chemotherapy (FLOT) will be administered post-surgery as per current standard protocol (see Figure 1).

The study population will be drawn from high-risk gastric cancer patients who are planned for curative multimodal treatment. The full inclusion and exclusion criteria are listed in Table 1.

**Table 1:** Inclusion and exclusion criteria for PIPAC-O+ protocol.

Inclusion	Exclusion
Adult patients (>18 years of age)	Extra-abdominal metastatic disease and established and proven peritoneal metastatic disease.
Clinical and histopathological confirmation of gastric adenocarcinoma.	Gastric outlet or bowel obstruction requiring nasogastric tube or percutaneous endoscopic gastrostomy
Undergoing or recommended for curative multimodal treatment for gastric adenocarcinoma	History of allergic reaction to platinum containing compounds
One or more of the following oncological high-risk features must be present: – Positive cytology in initial peritoneal lavage – Signet ring cell pathology – Diffuse type gastric adenocarcinoma – Linitis plastica – Proximal location – Serosal involvement of the stomach (T4) – High nodal disease burden – Young age at diagnosis (<50 years)	Severe renal impairment, myelosuppression, severe hepatic impairment, severe myocardial insufficiency, recent myocardial infarction and severe arrhythmias
Must have the ability to understand and sign a written informed consent form, which must be obtained prior to initiation of study procedures.	Immunocompromised patients such as those with an immunosuppressive medication or a known disease of the immune system
	Pregnancy/breast feeding
	Any chronic medical or psychiatric condition that in the option of the investigators would make the subject unsuitable for the study or prevent compliance with study protocol procedures.

### Intervention

This protocol proposes to use the standard of care pathway with addition of PIPAC-O+ procedure two weeks prior to gastrectomy.

1) Diagnosis of gastric cancer with endoscopy and biopsy
2) CT scan chest/abdomen/pelvis followed by diagnostic laparoscopy and peritoneal cytology to confirm absence of metastatic disease
3) Discussion of patient at multidisciplinary team meeting (MDT)
4) Discussion with patient for enrolment in trial if inclusion criteria met
5) Neoadjuvant systemic chemotherapy (four cycles of FLOT)
*Three weeks post ending of neoadjuvant chemotherapy*
6) Restaging CT scan chest/abdomen/pelvis
7) Patient is clinically assessed by surgeon and completes QoL-30.
*Four weeks post ending of neoadjuvant chemotherapy*
8) Patient undergoes laparoscopy with peritoneal biopsy and PIPAC-O
*Six weeks post ending of neoadjuvant chemotherapy*
9) Gastrectomy and lymphadenectomy
*Thirteen weeks post ending of neoadjuvant chemotherapy*
10) Adjuvant systemic chemotherapy (four cycles of FLOT)

### PIPAC-O+ procedure

The PIPAC procedure has been well described in the literature since the first clinical application by Professor Marc Raymond in 2011 [12, 13]. Briefly, general endotracheal anaesthesia is administered, and laparoscopic access is obtained. After insufflation of a 12 mmHg CO_2_ pneumoperitoneum, two balloon trocars (5 and 12 mm, Applied Medical, Rancho Santa Margarita, United States of America) are inserted into the abdominal cavity. A peritoneal lavage and aspiration of the lavage fluid for cytology is performed. The nebulizer (Capnopen; Gothia Medica, Billdal, Sweden) is connected to an injector pump and inserted via a balloon trocar. At this point in the procedure, all staff leave the operating theatre and the injector pump is activated from outside the room. The patient is remotely monitored throughout by the operating team. A pressurized aerosol containing Oxaliplatin 92 mg/m^2^, constituted in 150 mL dextrose is applied. Injection parameters are: flow of 0.5 mL/s, maximum upstream pressure of 200 psi. Therapeutic pressure of 12 mmH_2_O is maintained for 30 min at a temperature of 37 °C. Pneumoperitoneum is de-sufflated via a closed line into the air-waste system. Trocars are removed, and the procedure is concluded. Should rescue therapy be required, the administration will be stopped, and the anaesthetist will enter the theatre. Procedures will be enacted as per standard of care.

### Post PIPAC-O+ procedure

Participants will be admitted post-operatively and monitored as per standard of care. Once appropriate criteria have been met, they will be discharged from the hospital with a planned procedure date for gastrectomy. Any adverse events or complications that occur in hospital or post discharge will be recorded.

The patient will undergo oncological resection of the primary gastric cancer as per standard of care. Peritoneal lavage and aspiration cytology will be performed at the start of the procedure. Note will be made of any unusual findings during the procedure which are potentially due to PIPAC-O+. Postoperative surgical follow up as per standard of care and surgical team. The patient will begin post-operative chemotherapy as per guidelines.

Common Terminology Criteria for Adverse Events (CTACE) is commonly used in assessing adverse events of chemotherapy. The Clavien–Dindo Classification allows surgical complications to be assessed. Quality of Life questionnaires will be administered before and after the PIPAC-O+ procedure.

### Statistical considerations and data analysis

As the goal for this study is to demonstrate safety and feasibility, the primary endpoint is for the 10 consented patients to complete the protocol with no impact on oncological surgical procedure, surgical morbidity and adhering to the current standard pathway without delays or compromising the timelines. Univariate descriptive statistics and frequency distributions will be calculated, as appropriate for all variables. Baseline values for demographic, clinical and outcome variables (primary and secondary) will be tabulated for the treatment groups. These analyses will help identify potential confounding variables to be used as covariates in sensitivity analyses. Interim safety analyses will be performed once five patients have become evaluable.

The study will be conducted in full conformance with principles of the “Declaration of Helsinki”, Good Clinical Practice (GCP) and within the laws and regulations of the country in which the research is conducted. This study has CALHN HREC approval (13705).

## Outcomes and significance

The proposed intervention aims to reduce the risk of metastatic peritoneal disease in patients who have completed curative multimodal treatment for high risk gastric cancer. If shown to be safe and feasible, this treatment can be applied to a larger patient cohort. A prospective randomized study consisting of standard treatment arm and a standard treatment combined with PIPAC, respectively will be next step to evaluate the clinical benefit of the PIPEC.

## References

[1] Alberto M , Brandl A , Garg P , Gul-Klein S , Dahlmann M , Stein U , . Pressurized intraperitoneal aerosol chemotherapy and its effect on gastric-cancer-derived peritoneal metastases: an overview. Clin Exp Metastasis 2019;36:1–14. 10.1007/s10585-019-09955-4. 30715654

[2] Lu M , Yang Z , Feng Q , Yu M , Zhang Y , Mao C , . The characteristics and prognostic value of signet ring cell histology in gastric cancer: a retrospective cohort study of 2199 consecutive patients. Medicine 2016;95 e4052 10.1097/md.0000000000004052. 27399088 PMC5058817

[3] Al-Batran S , Homann N , Pauligk C , Goetze T , Meiler J , Kasper S , . Perioperative chemotherapy with fluorouracil plus leucovorin, oxaliplatin, and docetaxel versus fluorouracil or capecitabine plus cisplatin and epirubicin for locally advanced, resectable gastric or gastro-oesophageal junction adenocarcinoma (FLOT4): a randomised, phase 2/3 trial. Lancet 2019;393:1948–57. 10.1016/s0140-6736(18)32557-1. 30982686

[4] Struller F , Horvath P , Solass W , Weinreich F , Strumberg D , Kokkalis M , . Pressurized intraperitoneal aerosol chemotherapy with low-dose cisplatin and doxorubicin (PIPAC C/D) in patients with gastric cancer and peritoneal metastasis: a phase II study. Ther Adv Med Oncol 2019;11 1758835919846402 10.1177/1758835919846402. PMC6535725 31205501

[5] Gockel I , Jansen-Winkeln B , Haase L , Rhode P , Mehdorn M , Niebisch S , . Pressurized intraperitoneal aerosol chemotherapy (PIPAC) in gastric cancer patients with peritoneal metastasis (PM): results of a single-center experience and register study. J Gastric Canc 2018;18:379–91. 10.5230/jgc.2018.18.e37. PMC6310762 30607301

[6] Wagner A , Syn N , Moehler M , Grothe W , Yong W , Tai B , . Chemotherapy for advanced gastric cancer. Cochrane Database Syst Rev 2017;8 CD004064 10.1002/14651858.CD004064.pub4. 28850174 PMC6483552

[7] Cardona K , Zhou Q , Gonen M , Shah MA , Strong VE , Brennan MF , . Role of repeat staging laparoscopy in locoregionally advanced gastric or gastroesophageal cancer after neoadjuvant therapy. Ann Surg Oncol 2013;20:548–54. 10.1245/s10434-012-2598-6. 22941159

[8] Coccolini F , Cotte E , Glehen O , Lotti M , Poiasina E , Catena F , . Intraperitoneal chemotherapy in advanced gastric cancer. Meta-analysis of randomized trials. Eur J Surg Oncol 2014;40:12–26. 10.1016/j.ejso.2013.10.019. 24290371

[9] Kwon O , Chung H , Yu W . Early postoperative intraperitoneal chemotherapy for macroscopically serosa-invading gastric cancer patients. Canc Res Treat 2014;46:270. 10.4143/crt.2014.46.3.270. PMC4132443 25038762

[10] Garg PK , Jara M , Alberto M , Rau B . The role of pressurized intraperitoneal aerosol chemotherapy in the management of gastric cancer: a systematic review. Pleura Peritoneum 2019;4:20180127. 10.1515/pp-2018-0127. 31198852 PMC6545873

[11] Mortensen M. . Adjuvant PIPAC in gastric cancer patients (PIPAC-OPC4) 2019. Available from: https://clinicaltrials.gov/ct2/show/record/NCT04047004 ..

[12] Solass W , Kerb R , Mürdter T , Giger-Pabst U , Strumberg D , Tempfer C , . Intraperitoneal chemotherapy of peritoneal carcinomatosis using pressurized aerosol as an alternative to liquid solution: first evidence for efficacy. Ann Surg Oncol 2014;21:553–9. 10.1245/s10434-013-3213-1. 24006094 PMC3929768

[13] Reymond M , Solass W , Nadiradze G , Horvath P , Königsrainer A . Pressurized intraperitoneal aerosol chemotherapy (PIPAC). In: Fong Y , Gamblin T , Han E , Lee B , Zager J , editors. Cancer regional therapy, HAI, HIPEC, HILP, ILI, PIPAC and beyond. Cham: Springer; 2020:235–6. 10.1007/978-3-030-28891-4_20.

